# Engineering Parameters in Bioreactor's Design: A Critical Aspect in Tissue Engineering

**DOI:** 10.1155/2013/762132

**Published:** 2013-08-05

**Authors:** Nasim Salehi-Nik, Ghassem Amoabediny, Behdad Pouran, Hadi Tabesh, Mohammad Ali Shokrgozar, Nooshin Haghighipour, Nahid Khatibi, Fatemeh Anisi, Khosrow Mottaghy, Behrouz Zandieh-Doulabi

**Affiliations:** ^1^Department of Chemical Engineering, Faculty of Engineering, University of Tehran, P.O. Box 11365-4563, Tehran, Iran; ^2^Department of Biomedical Engineering, Research Center for New Technologies in Life Science Engineering, University of Tehran, P.O. Box 14395-1374, Tehran, Iran; ^3^Institute of Physiology, Medical Faculty, RWTH Aachen University, 52074 Aachen, Germany; ^4^National Cell Bank, Pasteur Institute of Iran, P.O. Box 1316943551, Tehran, Iran; ^5^Gustav Mahlerlaan 3004, 1081 LA Amsterdam, The Netherlands

## Abstract

Bioreactors are important inevitable part of any tissue engineering (TE) strategy as they aid the construction of three-dimensional functional tissues. Since the ultimate aim of a bioreactor is to create a biological product, the engineering parameters, for example, internal and external mass transfer, fluid velocity, shear stress, electrical current distribution, and so forth, are worth to be thoroughly investigated. The effects of such engineering parameters on biological cultures have been addressed in only a few preceding studies. Furthermore, it would be highly inefficient to determine the optimal engineering parameters by trial and error method. A solution is provided by emerging modeling and computational tools and by analyzing oxygen, carbon dioxide, and nutrient and metabolism waste material transports, which can simulate and predict the experimental results. Discovering the optimal engineering parameters is crucial not only to reduce the cost and time of experiments, but also to enhance efficacy and functionality of the tissue construct. This review intends to provide an inclusive package of the engineering parameters together with their calculation procedure in addition to the modeling techniques in TE bioreactors.

## 1. Introduction

Tissue engineering aims to generate three-dimensional (3D) artificial tissues. Its consequential task is to regenerate human tissue or develop cell-based substitutes for tissue in order to restore, reconstruct, or improve tissue functions [1, 2]. Achieving biological and mechanical functionality of the newly formed tissue is paramount for tissue engineered structures. Yet current research often focusses on form rather than function. Regeneration of functional organs demands intensive researches and studies in every aspect of TE [3], since creating a functional tissue requires the efficient growth of various types of cells on a single 3D structure [2].

Bioreactors can aid the production of functional 3D tissues as follows: (1) by maintaining a desired uniform cell concentration within the scaffold during cell seeding, (2) by controlling microenvironmental parameters (e.g., temperature, pH, pressure, oxygen tension, metabolites, regulatory molecules, shear stress, and electrical pacing) and aseptic parameters (e.g., feeding, waste removal, and sampling), (3) by facilitating mass transfer [4–7], and more importantly (4) by allowing for automated processing steps. 

Moreover, each special type of tissue structure and production procedure (e.g., skin, bone, blood vessel, cartilage, and myocardium) necessitates a unique kind of bioreactor design which requires both biological and engineering conditions to be addressed along with reliability, reproducibility, scalability, and safety issues [8–10].

In this review, key technical challenges between biological parameters and engineering parameters are recognized along with an overview of present mathematical modeling and monitoring of tissue growth carried out in the Research Center for New Technologies in Life Science Engineering at University of Tehran (UTLSE) to help deal with ongoing challenges.

## 2. Engineering Parameters in TE Bioreactor Design

Generally, the major responsibilities of a bioreactor are to provide a biomechanical and a biochemical environment that controls nutrient and oxygen transfer to the cells and metabolic products from the cells [11–13]. Mass transfer problems (e.g., oxygen and nutrient supply and removal of toxic metabolites) must always be taken into account. The size of most engineered tissues is limited as they do not have their own blood system and the cells are only nourished by diffusion [14, 15]. Since tissue constructions should have larger dimensions to become functional, mass transfer limitation can be considered as one of the greatest engineering challenges [1].

Moreover, biomechanical stimuli such as shear stress can be applied throughout the bioreactor by means of culture medium flow [10, 16]. In this condition, nutrient and waste transfer are automatically regulated by the flow of the growth medium. Other types of mechanical stimuli can also be applied to tissue constructs using a bioreactor, including axial compression or tensile forces [11].

Although biomechanical stimuli have many advantages for tissue engineering, mechanical stimuli can also induce tissue degradation, by alterations in the synthesis of matrix [16]. All in all, the response of some types of cells to mechanical stress causes radical changes to the tissue structure and composition which leads to alterations in tissue functionality.

In the following section, some of the engineering parameters which help providing physical stimulation to TE constructs in order to enhance tissue formation and their concomitant challenges are specified.

### 2.1. Mass Transfer through Bioreactors

The major obstacle that hinders practical application of 3D cell seeded constructs is mass transfer [5]. After distributing cells throughout porous scaffolds, a key challenge is the maintenance of cell viability, especially within the interior of the construct during prolonged culture. Nutrients, oxygen, and regulatory molecules have to be efficiently transferred from the bulk culture medium to the tissue surfaces (i.e., external mass transfer) as well as to the interior cells of the tissue construct (i.e., internal mass transfer). In addition, metabolites and CO_2_ are to be removed from the cells within the tissue to the bulk medium. While external mass transfer rates depend primarily on hydrodynamic conditions in a bioreactor, internal mass transfer rates may depend on a combination of diffusion and convection mechanisms (typically induced by medium perfusion or scaffold deformation). Internal mass transfer depends strongly on the scaffold's structure and porosity, the overall cell or scaffold construct size, and the diffusion rate through the biomaterial [17, 18].

Improving the scaffold design will aid efficient mass transfer. For example, a laminar flow within tubular structures located within a scaffold may be beneficial for the generation of large TE constructs but requires the development of advanced bioreactor systems.

Amongst mass transfer mechanisms stated previously, oxygen transfer is a matter of the utmost importance due to poor solubility of oxygen in culture medium [9, 19, 20]. In addition, the diffusive penetration depth of oxygen within tissues *in vivo* is in the range of only 100 to 200 *μ*m [19]. Thus, maintaining the balance between oxygen delivery to cells and their oxygen consumption is critical, considering this diffusive distance. Therefore, the oxygen tension adjustment is a critical matter in the design process of any bioreactor [21].

In applications germane to TE, the oxygen demand will fluctuate each time. During the initial expansion phase, cell density increases with time, and consequently, the overall demand for oxygen also increases. Cells may change from a proliferative state to the state of differentiation during the later stages of the culture. This change has implications for oxygen transfer, since proliferating cells typically have a higher oxygen demand per cell than differentiating cells [2]. Therefore, during the differentiation phase, the oxygen demand is likely to decline gradually.

A culture can be aerated by one, or a combination, of the following methods: surface aeration, direct sparging, indirect and/or membrane aeration (diffusion), medium perfusion, increasing the partial pressure of oxygen, and increasing the atmospheric pressure [22]. The transport of dissolved oxygen in a bioreactor occurs in three regions as follows:

- bulk fluid phase of the bioreactor (global mass transfer),
- from the bulk to the surface of the aggregated cells (internal mass transfer),
- through the aggregated cells (external mass transfer).

In the first step, at the gas-liquid interface, the rate of oxygen entering the medium is limited by the relatively low solubility of oxygen in aqueous medium. The scalar concentration distributions in the vessel for the global mass transfer depend on the flow field of the vessel and the net rate of consumption or production [23, 24]. Therefore, the oxygen concentration in the fluid experienced by the cells is a result of the balance between the oxygen delivery across the medium layer called the oxygen transfer rate (OTR) and the rate of oxygen consumption by cells named the oxygen uptake rate (OUR). Therefore, the oxygen concentration can be ten times lower as one would anticipate based on the equilibrium within the gas phase [25]. Oxygen availability has vigorous effect on cell culturing kinetics. For instance, increasing the amount of dissolved oxygen (DO) which can be done by increasing the OTR may lead to improve secondary metabolism too. The rate of OTR highly affects the liquid phase mass transfer coefficient (*k*_*L*_ *a*) and, then, the productivity. Therefore, it is essential to determine the DO level in the bioreactor [24, 26–28]. 

There are different methods for assessing the amount of oxygen delivered from the air to the culture environment (Table 2). The sulfite system is one in which transformed oxygen content from air to the aqueous solution is determined by means of the oxidation of sodium sulfite to sodium sulfate by oxygen. It could characterize the completing point of the reaction by means of a pH indicator, since the sulfate ions have more acidic activity than the sulfite ones. This method was applied in the presence of cobalt catalyst for determining the OTR and for studying the function of a perfusion bioreactor designed by UTLSE. It was concluded that oxygen delivery is appropriate and the bioreactor readily supplies the minimum required oxygen of the various cells. By considering the calculated OTR_max⁡_ of 0.012 mol/L/hr and the largest *k*_*L*_ *a* = 0.02 L/s, calculations showed that bioreactor supplies the required oxygen of culturing more than 10^10^ CHO cells in the 80 mL culturing volume [29]. 

### 2.2. Mechanical Stimulation

The field of TE gradually recognizes the importance of mechanical stimuli (e.g., mechanical compression, mechanical stretch, hydrodynamic pressure, and fluid flow) in the maturation of organs [5]. Mechanical stimulation is one of particular interest for musculoskeletal tissue engineering, cartilage formation, and cardiovascular tissues [30–35]. Mechanical interactions during tissue growth, between different components, that is, cells, water, and scaffold material, can determine whether cells form cell aggregates or disperse throughout the scaffold [36–38]. Selection of optimal physical parameters is complicated by a variety of cell types, scaffolds, forces, applied regimes, and culture medium available. 

Cells in aggregates are exposed to higher shear stresses than single cells due to their large particle diameter [39]. It is widely accepted that shear stress has a dominant impact on tissue function and viability. Different values are reported for the maximal sustainable shear stress for different types of cells [40, 41]. Indeed, high shear stress on the surface of the scaffold, caused by a flow of fluid, can peel off attached cells and in this condition, tissue growth is significantly slower compared with static cultures.

Simply, orientation and function of the cells is affected by fluid flow shear stress. Shear stress is a particular interesting stimulus for mammalian cell cultures because many cell types are responsive to shear stress [42–45]. For instance, it was observed that shear stress affected endothelial cell proliferation and oriented them toward flow direction [31]. There are many qualitative means for investigating fluid flow, which are summarized in Table 3.

In addition, the secretion of biological factors by stem cells can be increased by biomechanical forces. Therefore, it is important to acquire an understanding of the mechanisms by which hemodynamic forces are detected and converted into a sequence of biological responses within the cells [46]. For instance, changes in pressure or shear stress induce the rapid release of nitric oxide (NO) from the vascular endothelium [47–49]. Studies at UTLSE in a simple parallel plate flow chamber showed that NO production by Human umbilical vascular endothelial cells (HUVECs) is fluid shear stress rate dependent (data not shown).

In fact, the determination of how mechanical forces can be utilized is a challenge for bioreactor design in order to reach the proper environment necessary to produce the desired tissue engineered product. Pulsatile perfusion bioreactors integrated with elastic polymeric scaffolds enhance development and differentiation of small tissue engineered blood vessels [50–53]. Furthermore, custom-designed bioreactors utilizing biaxial strain for the mechanical stimulation of skeletal tissues were developed [54, 55].

### 2.3. Electrical Stimulation

In addition to mechanical stimuli commonly arising in tissue engineering context, electrical stimulation or even combined approaches incorporating electrical/mechanical cues need to be provided *in vitro* for obtaining an appropriate functionality of engineered tissue. Electrical stimuli are currently mainly applied in the field of cardiac tissue engineering to regenerate the infarcted area after heart failure [56, 57]. Radisic et al. [58] showed that electrical waves in a square form with frequency of 1 Hz and power of 5 V/cm can induce contractile properties in cardiac TE constructs. The disruption of regularity of ions in an electrically affected construct leads to redistribution of charge which can then alter the pH gradient in the media which can be used to tailor specifically enhanced cellular function [59]. Finally, electrical pacing associated with mechanical cues in the culture when applied to the electrospun cardiac constructs resulted in better alignment, elongation, and upregulation of cardiac proteins compared with static cultures [60]. 

## 3. Comparison between Different Types of TE Bioreactors Based on Engineering Parameters 

Bioreactors that are currently widely used in TE are static and mixed flasks, rotating wall, and perfusion bioreactors. These bioreactors offer three distinct flow conditions (static, turbulent, and laminar), and hence a different rate of nutrient supply to the surface of tissue construct [24]. They also differ in mass transfer and shear stress rates experienced by the cultured cells.  Table 1 compares engineering parameters of different TE bioreactors.

Although* static culture* is simply designed and operated, there are nutrient diffusion limitations with large constructs since both external and internal mass transfer are undertaken by diffusion [9, 11, 22]. Statically cultured constructs often have a heterogeneous structure and composition, including a necrotic central region and dense layers of viable cells encapsulating the construct outer edge [17]. This condition appears due to concentration gradients, with local depletion of nutrients and accumulation of waste materials [18].

Cell survival and assembly on many surfaces of engineered tissues can be improved by construct cultivation in *stirred flask* bioreactors [61–65]. Within such flasks, scaffolds are attached to needles hanging from the lid of the flask for dynamic seeding. Convective flow, generated by a magnetic stirrer bar, allows continuous mixing of the medium surrounding the construct [24]. This environment improves nutrient diffusion and promotes cell proliferation throughout the constructs in comparison to static condition. However, the shear forces acting on the constructs are heterogeneous, which prevents homogenous tissue development [11].

In order to enhance external mass transfer under a laminar flow condition, the tissue engineered constructs can be cultivated in *rotating wall *bioreactors [63, 65–67]. Dynamic laminar flow of rotating bioreactors generally improves properties of the peripheral tissue layer. Also, in such bioreactors, no fibrous capsule is formed, but the limitations of the diffusional transfer of oxygen to the construct interior still remain [24]. As compared to the turbulent flow within stirred flasks, the dynamic laminar flow in rotating wall vessels contributes to reduced levels of shear stress experienced by cells on the construct. Amongst other, this aides the formation of cartilaginous tissues containing higher amounts of more uniformly distributed glycosaminoglycans (GAG) and collagen [18, 68].

In addition, a key point to note is that convective transfer around and through an engineered tissue at the proper flow rate can dissipate gradients of nutrients and maintain tissue mass [69]. In a novel strategy, Yu et al. [70] mixed microspheres of different densities in order to vary and modify flow velocity within a scaffold through the rotating wall bioreactor. Compared to static three-dimensional controls, culturing rat primary calvarial cells under dynamic flow conditions in a rotating system reveals a more uniform distribution of cells in the scaffold interior and also enhances phenotypic protein expression and recuperates mineralized matrix synthesis. In addition, Zhang et al. [71] recognized that scaffolds seeded by human fetal mesenchymal stem cell (hfMSC) reached cellular confluence earlier with greater cellularity and also conserved high cellular viability in the core of them compared to a static culture.

*Perfusion *bioreactors are used in order to force culture medium through the pores of solid porous 3D scaffolds, thereby enhancing nutrient transport and providing mechanical stimuli to the cells (e.g., [63, 65, 101–104]). In such systems, oxygen and nutrients are supplied to the construct interior by both diffusion and convection. The flow rate can be optimized with respect to the limiting nutrient, which is mostly oxygen due to its low solubility in culture medium [24, 105]. Perfusion of chondrocyte-seeded scaffolds was reported to elevate GAG synthesis and retention within the extracellular matrix (ECM) [106], as well as a uniform distribution of viable human chondrocytes. A perfusion system can provide a well-defined physicochemical culture environment which has great potential to generate cartilage grafts [68] or vascular grafts of clinically relevant size [107, 108]. Bioreactors that perfuse the culture medium directly through the pores of a scaffold enhance mass transfer rate not only at the construct periphery but also within the internal pores. This can potentially eliminate mass transfer limitations. Perfusion bioreactors can offer greater control of mass transfer than other conventional systems but the potential for flow to follow a preferential path through the construct still remains a problem. This phenomenon happens particularly for scaffolds with a wide pore size distribution and nonuniformly developing tissues, leaving some regions poorly nourished, while others are perfused strongly.

It is confirmed that cartilage-like matrix synthesis by chondrocytes, chondrocyte growth, and differentiation and deposition of mineralized matrix by bone cells are enhanced by direct perfusion bioreactors [109]. It is worth to notice that the flow rate in the microenvironment of cells is to a great extent responsible for the changes of medium perfusion. Therefore, to optimize a perfusion bioreactor for tissue engineering applications, the balance between the extent of nutrient supply, the transport of metabolites to and away from cells, and the fluid-induced shear stress effects on cells located at the surface and in the porous structures of the scaffold should be considered [17, 21, 105].

In order to gain a better understanding on how physical factors modulate tissue development, it is necessary to integrate bioreactor studies with quantitative analyses and computational modeling of changes in mass transfer and physical forces experienced by cells [17].

## 4. Mathematical Modeling of Engineering Parameters

Mathematical modeling in terms of fundamental physical and biochemical mechanisms can be used to justify experimental results and determine future research directions [110–113]. Relatively few mathematical modeling studies have focused on bioreactor culture of cell-seeded porous structures for TE [114, 115].

In the first stage, numerical simulation plays an important role in prediction of the global dynamic response in different parts of bioreactors. Moreover, numerical evaluation provides insight into local hydrodynamic changes in tissue constructs in order to generate quantitative anticipation of the tissue development within a bioreactor system [116]. Finally, with the aid of recently available computational tools, variables (e.g., flow fields of a particular bioreactor design [117, 118], incorporation of the mechanics of the scaffold material [119], and the sufficiency of bioreactor cultures [117, 120, 121], shear stresses and mass transfer in scaffold-containing bioreactors [118, 122]) can be estimated.

As an example, Sengers et al. [3] in their review concentrated on the contribution of computational modeling as a framework to obtain an integrated understanding of key processes including nutrient transfer, matrix formation, dynamics of cell population, cell attachment and migration, and local mutual interactions between cells.

### 4.1. Nutrient and Mass Transfer

The amount of delivered oxygen is a significant factor in designing the cell culture bioreactors. One major obstacle preventing proper understanding of oxygen tension in TE constructs is a lack of mathematical models that can predict which parameters are beneficial for avoiding oxygen limitation and increasing oxygen diffusion across serial resistances [114, 118, 121]. This problem was resolved at UTLSE by applying traditional convective mass transfer models combined with Maxwell-Stefan diffusion mass transfer equation.

**Figure 1:**
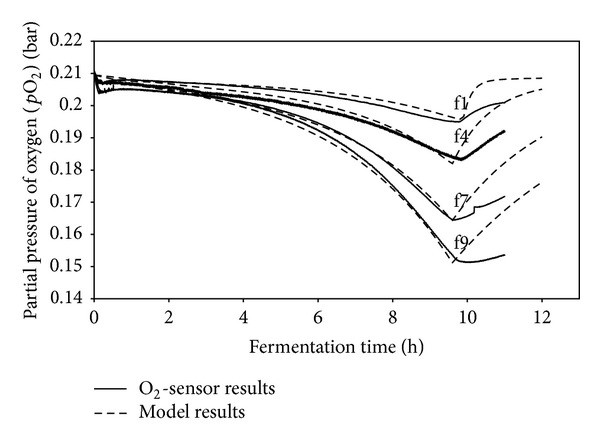
Comparison between unsteady state model and experimental results for the partial pressure of oxygen in the headspace of the ventilation flasks f1, f4, f7, f9 (sterile plug dimensions in f1 < f4 < f7 < f9) is obtained for the fermentation of *C. glutamicum *DM 1730 on 10 g/L glucose and 21 g/L MOPS (*V*_*L*_ = 10 mL, *n* = 400 rpm, *T* = 30°C, *d*_*o*_ = 5 cm, *Y*_*x*/*s*_ = 0.48, *Y*_*x*/*o*_2__ = 53 g/mol, RQ = 1 where *d*_*o*_, *V*_*L*_, *Y*_*x*/*s*_, *Y*_*x*/*o*_2__, and RQ are shaking diameter, filling volume, yield of biomass with respect to substrate, yield of biomass with respect to oxygen, and respiration quotient, resp.).

The reliability of the model can be examined by comparing the model results obtained with sulfite experiments done with four geometries of shake flasks (Figure 1).

As can be seen from Figure 1, the value for the *p*O_2_ is 0.2095 bar at the onset of the experiment. Oxygen partial pressure decreases over time as the chemical reaction proceeds. The flasks with the greater sterile plug dimensions represent lower mass transfer rates which resulted from hindered diffusion. This gives rise to a lower partial pressure of oxygen [84, 85, 123, 124].

Yan et al. [114] developed a novel mathematical model to represent the glucose and oxygen distribution and the cell growth in a 3D cell-scaffold construct in a perfusion bioreactor. Numerical methods are employed to solve the equations involved, with a focus on investigating the effect of various factors such as culturing time, porosity, and flow rate, which are controllable in the scaffold fabrication and culturing process, on cell cultures.

Along these lines, Pisu et al. [125] proposed an improved description of oxygen consumption and GAG production by bovine chondrocytes, which is thoroughly related to cellular metabolism. The latter is simulated through appropriate population balance models which include cellular anabolic and catabolic rates.

Abdollah and Das [126] presented a general modeling framework to characterize nutrient (oxygen and glucose) transfer in a hollow fiber membrane bioreactor (HFMB) for bone tissue growth. The framework relied on solving coupled Navier-Stokes and the Maxwell-Stefan convection-diffusion-reaction equations. It is indicated that due to multicomponent interactions, mass severe transfer limitations may arise severely when inlet concentration of nutrients, molecular size of the solutes, and wall membrane thickness are increased.

Rivera-Solorio and Kleis [23] used a mathematical model to investigate the local mass transfer of dissolved oxygen to the surface of freely suspended cell aggregates in a bioreactor operating in microgravity. They simulated the mass transfer in systems in which cultured cells are attached to small microcarriers in a rotating bioreactor in simulated and real microgravity.

Also, Yu et al. [127] evaluated oxygen transfer in a microbioreactor for animal cell suspension culture using the commercial software Fluent. They proposed two correlations in order to calculate the liquid-phase oxygen transfer coefficient and the minimum oxygen concentration in a microbioreactor, to provide insight into choosing the proper operating parameters in animal cell culture.

### 4.2. Fluid Flow

To better realize the effect of fluid flow during tissue regeneration, a number of studies using computational fluid dynamic (CFD) have been accomplished [128–134]. These CFD studies revealed detailed profile of pressure, velocity, flow fields, shear stresses, and oxygen transfer in tissue culturing chambers of various bioreactor designs. This is very useful for the design optimization of internal geometric configurations of bioreactors [116]. 

Lawrence et al. [16] explored the effect of reactor geometry on flow fields using the computational fluid dynamics software Comsol Multiphysics 3.4. The Brinkman equation was used to model the permeability characteristics within the chitosan porous structure. Results showed significant increase in pressure with reduction in pore size, which could limit the fluid flow and nutrient transport.

Subsequently, flow characteristics are analyzed using either Darcy's equation [135] or the Brinkman equation considered as an extension of Darcy's equation. The Brinkman equation accounts for both viscous and drag forces in the porous medium. It can be reduced to either Navier-Stokes equation or Darcy's law if forces become dominant. The Brinkman equation is as follows [16]:

(1) $$\begin{array}{c} \mu \nabla^{2}u_{s}-\frac{\mu }{k}u_{s}=\nabla p,\;\;\nabla u_{s}=0, \end{array}$$

where *k* the permeability of the porous medium, *u*_*s*_ denotes the fluid superficial velocity vector, *p* the fluid pressure, and *μ* is the effective viscosity in the porous medium. Nonporous sections of a bioreactor were modeled as incompressible Navier-Stokes regions. The permeability of the porous medium (*k*) is a geometric characteristic of the porous structure at several length scales. The Navier-Stokes equation together with continuity equation provides an essential tool to investigate the mechanical behavior of fluid in shaken bioreactors. 

**Figure 2:**
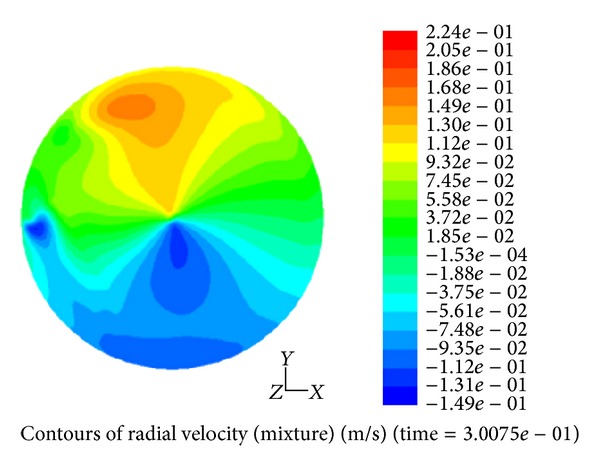
Radial velocity distribution in a shaken 24-wells bioreactor that illustrates inhomogeneous map of radial velocity at the interface of liquid and air.

**Figure 3:**
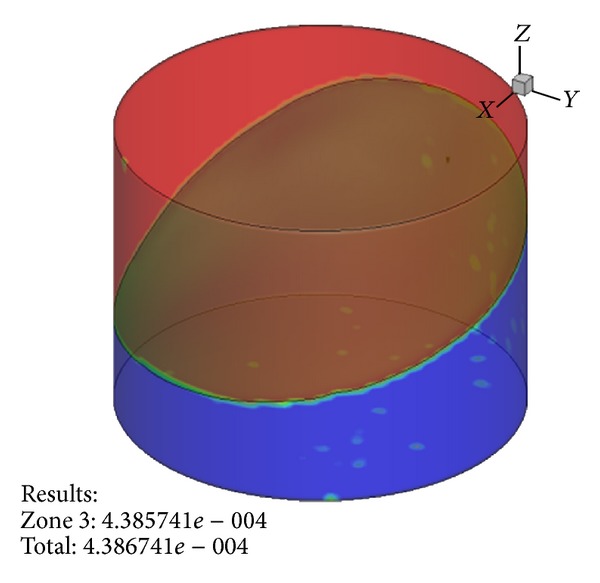
Volume fraction distribution in a shaken 24-wells bioreactor that allows accurate prediction of gas-liquid interface area within the shaking bioreactor.

Our research center began intensive studies of hydrodynamics applying CFD in shaken microbioreactors including 24-well plates, shaken at various shaking frequencies. For instance, schematics of the liquid phase fraction and radial velocity profiles at 0.7 cm distance from the bottom plane are shown in Figures 2 and 3 at shaking frequency of 200 rpm. 

Output data from phase fraction simulation gave insight in the gas-liquid interfacial surface area, which then helps to determine the exact mass transfer coefficient (*k*_*L*_ *a*) values. Furthermore, the mean radial velocity at the interface provides a guideline for obtaining wall shear stress within the entire domain of the bioreactor. 

The outcomes of shear stress simulation experiments confirm that the magnitude of this mechanical quantity rarely exceeds 1 Pa at the bottom of the plate. This value of shear stress can be withstood by most mammalian cells [136].

**Figure 4:**
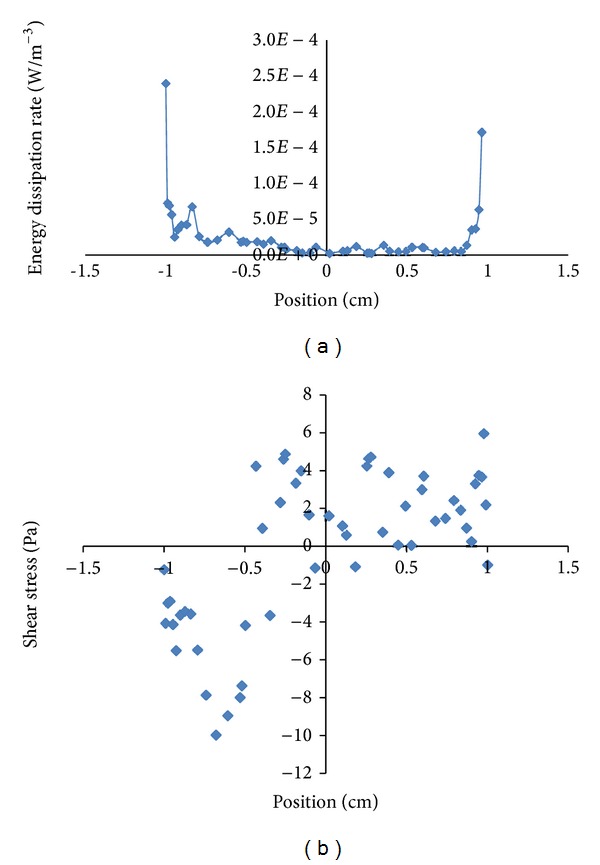
Energy dissipation rate (a) and shear stress distribution (b) versus radial position on a scaffold with radius of 1 cm that obviously represents safe generated shear stress on the scaffold for mammalian cell cultures.

In addition, a novel flow chamber was developed in our research center to assess the effect of fluid flow on the efficiency of nutrient transport and the endothelial cell stability. This chamber exhibits the major features of a standard parallel flow bioreactor in which a circular silicon scaffold is centrally located. To accomplish this, CFD was used to discretize mathematical equations. Energy dissipation rate (EDR) and shear stress were plotted versus position in the cylinder at a flow rate of 75 mL/min (Figure 4).

For either plots of EDR and shear stress, a symmetrical pattern reveals that a homogeneous distribution of these mechanical characteristics of flow exists. However, for EDR data, some values deviate slightly between both sides of the cylinder because of a flow maldistribution which is due to a mild turbulence over the scaffold. 

**Figure 5:**
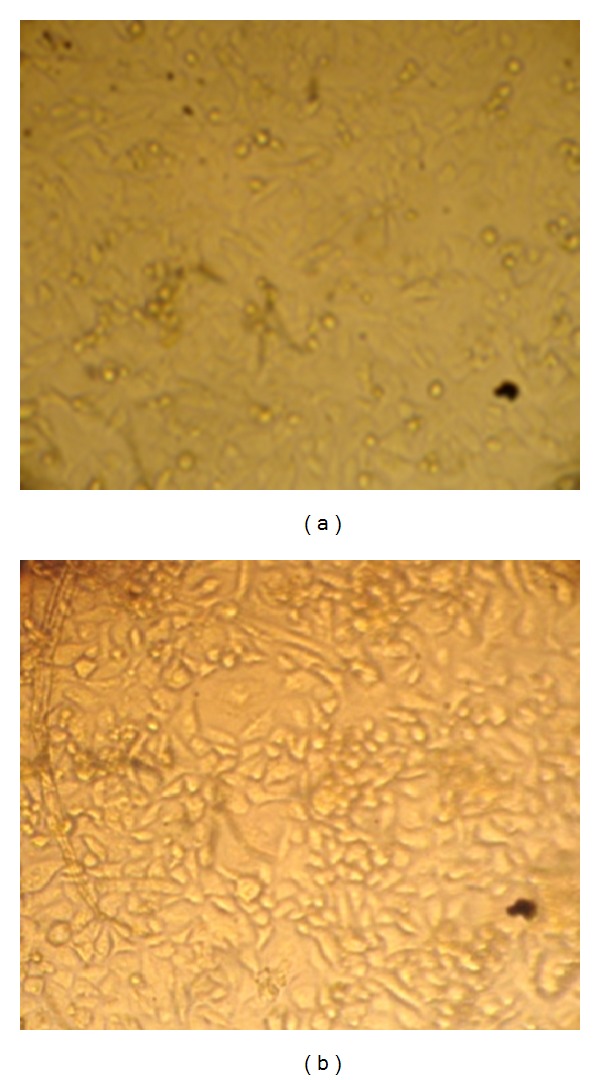
Schematics of cell morphology (a) before (b) after initiation of flow indicating flow assisted elongation of cells under continuous flow.

After completion of simulations, cell experiments were conducted for a 1 hr period. These experiments showed that at a volumetric flow rate of 75 mL/min, the cell viability and stability are maintained, but no specific cell orientation is present (Figure 5).

In general, computational fluid dynamics applications in bioreactor development can be extended to new designs such as a novel perfusion bioreactor developed at UTLSE.

**Figure 6:**
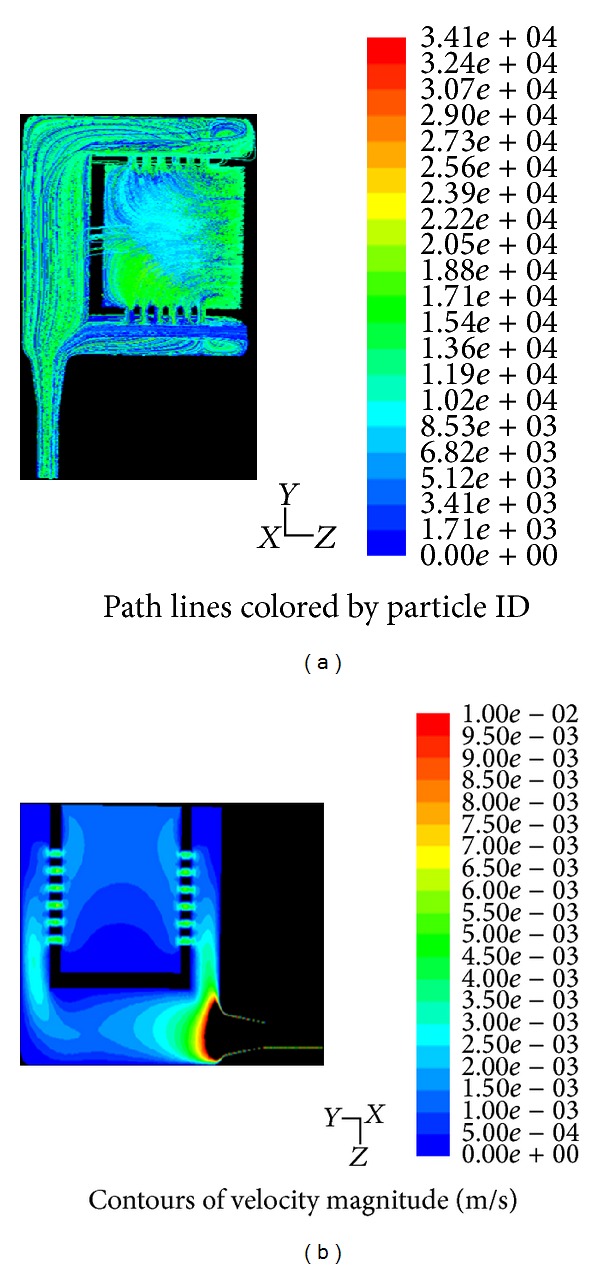
Demonstration of path lines to track the fluid particles within the bioreactor (a) and velocity magnitude to evaluate maximal shear stress in order to optimize shear stress distribution (b) in a perfusion bioreactor belonging to UTLSE.

In order to assess mechanical as well as oxygen characteristics of this novel perfusion bioreactor, scientists at UTLSE used CFD to determine fluid velocity as well as pathlines features of the flow.  Figure 6 further describes the computational attributes of the system. 

The initial approximation of fluid flow dynamics attained with CFD is extremely beneficial in reducing time and costs of development of the bioreactor [29].

Yu et al. [137] applied a CFD model to simulate the flow and oxygen concentration fields in a microbioreactor, in which a small magnetic bar was placed in a culture well to enhance the medium mixing. It was found that the hydrodynamic environment could be appropriate for animal cell culture when the microbioreactor operated at a stirrer rotating speed of 300 rpm and working volume of 4 mL.

Bilgen and Barabino [138] took advantage of CFD modeling to characterize the complicated hydrodynamic environment of a wavy-walled bioreactor applied for cultivation of tissue-engineered cartilage structures. They also analyzed the changes in the flow field when TE constructs are present in the bioreactor. The flow-induced shear stress experienced by engineered constructs cultivated in the wavy walled bioreactor was much lower than that of spinner flask. The radial or axial position of the constructs can modulate this shear stress.

Lawrence et al. [16] used rectangular and circular bioreactors with three different inlet and outlet paradigms. By the use of CFD, geometries were simulated in two cases, with and without the presence of a porous structure. Residence time distribution analysis using the change of a tracer within a bioreactor revealed nonideal fluid distribution characteristics. The result represented a significant increase in pressure with a decrease in pore size, which could lead to low fluid flow and nutrient transfer limitation.

### 4.3. Cell Growth, Proliferation, and Viability

Chung et al. [111] developed a mathematical model for the static culture of cells grown on porous scaffolds. Results showed that the overall cell growth allows cells to spread more uniformly, while it prevents cells from competing for nutrients at the same site. They then described a mathematical model to examine the effects of medium perfusion on the cell-scaffold constructs [120]. They proposed a three-layer model, highlighting the enhancement of cell growth by medium perfusion. The model is quite detailed, involving a cell construct sandwiched between two fluid layers in order to mimic the culturing environment of direct perfusion. Although the model is valuable in developing engineered cell constructs, the enormous number of essential formulas and boundary conditions make the model cumbersome. Therefore, a compact mathematical model was to describe cell growth within a porous scaffold under direct perfusion. Neglecting the two fluid regions sandwiching the scaffold, the model contains only the scaffold region for computational purposes [110]. 

Shakeel [118] in his thesis developed a model which describes the key features of the tissue engineering processes such as the interaction between the cell growth, variation of material porosity, flow of fluid through the material, and delivery of nutrients to the cells. The fluid flow through the porous scaffold and the delivery of nutrients to the cells was modeled by Darcy's law and the advection-diffusion equations, respectively. For modeling the cell growth, a nonlinear reaction diffusion system was used. The results show that the distribution of cells and total cell number in the scaffold depends on the initial cell density and porosity of the scaffold.

 A unique set of dynamical mathematical models was used to accurately predict metabolite and cell concentration in an aerated miniaturized shaking bioreactor at UTLSE. The major advantage of such a mathematical model is that it provides a robust tool to solve complicated oxygen transfer which unfavorably hampers metabolite production in bioprocesses. 

The combination of equations which make a link between liquid phase oxygen concentration and rate of oxygen uptake with governing equations of cell concentration should be primarily solved to attain oxygen transfer rate (OTR) with respect to the course of time. 

**Figure 7:**
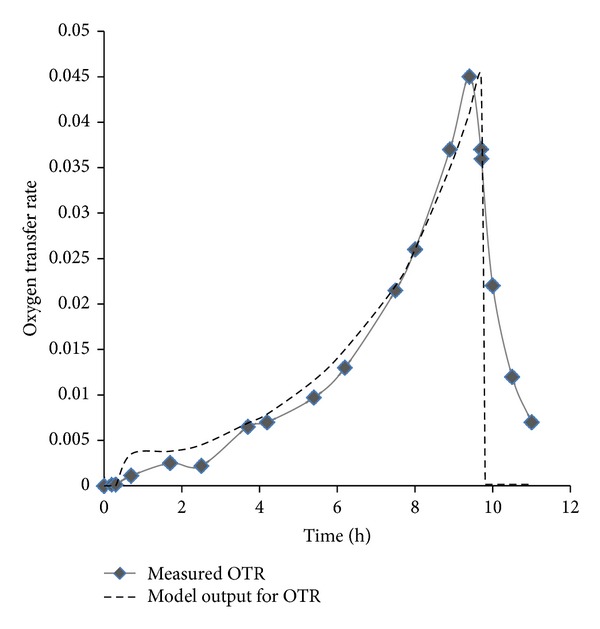
Comparison of OTR resulting from model and from experiments for a specific aerobic microorganism. The plot provides evidence of the proximity of OTR values between experimental and simulation results and of the efficacy of the simulation efforts.

Figure 6 suggests that as the model microorganism is undergoing accelerating growth, the oxygen transfer rate increases until the growth is inhibited and consequently the OTR falls down significantly.  Figure 7 illustrates the comparison between the model and experimental results for a model microorganism, which suggest that a minor discrepancy between their model and the results exist [139].

## 5. Conclusion

Engineering parameters occurring in a bioreactor are of equal importance as biological parameters and should therefore be investigated thoroughly in order to optimize outcomes of TE strategies. Internal and external mass transfer (e.g., oxygen, nutrient, and waste materials transfer) as well as mechanical stimulation (e.g., fluid flow and shear stress) should be monitored online. Between different types of bioreactors, the “Perfusion Bioreactor” is the most convenient for animal cell cultures on a solid porous scaffold. Perfusion bioreactors offer both convection and diffusion and can provide nearly *in vivo* physiochemical and environmentally stimuli for engineered tissue constructs. 

The operating conditions for diverse bioreactors can be very different per experiment. Therefore, it is essential to use mathematical equations and modeling techniques to simulate the optimal operating conditions in order to predict the best outcomes. Using the Brinkman equation along with powerful CFD codes can provide for investigating the effects of engineering parameters on the outcome of biological experiments. In this way, the efficacy of bioreactors, which is very low at present, can be optimized.

## Figures and Tables

**Table 1:** Comparison of engineering parameters in different TE bioreactors.

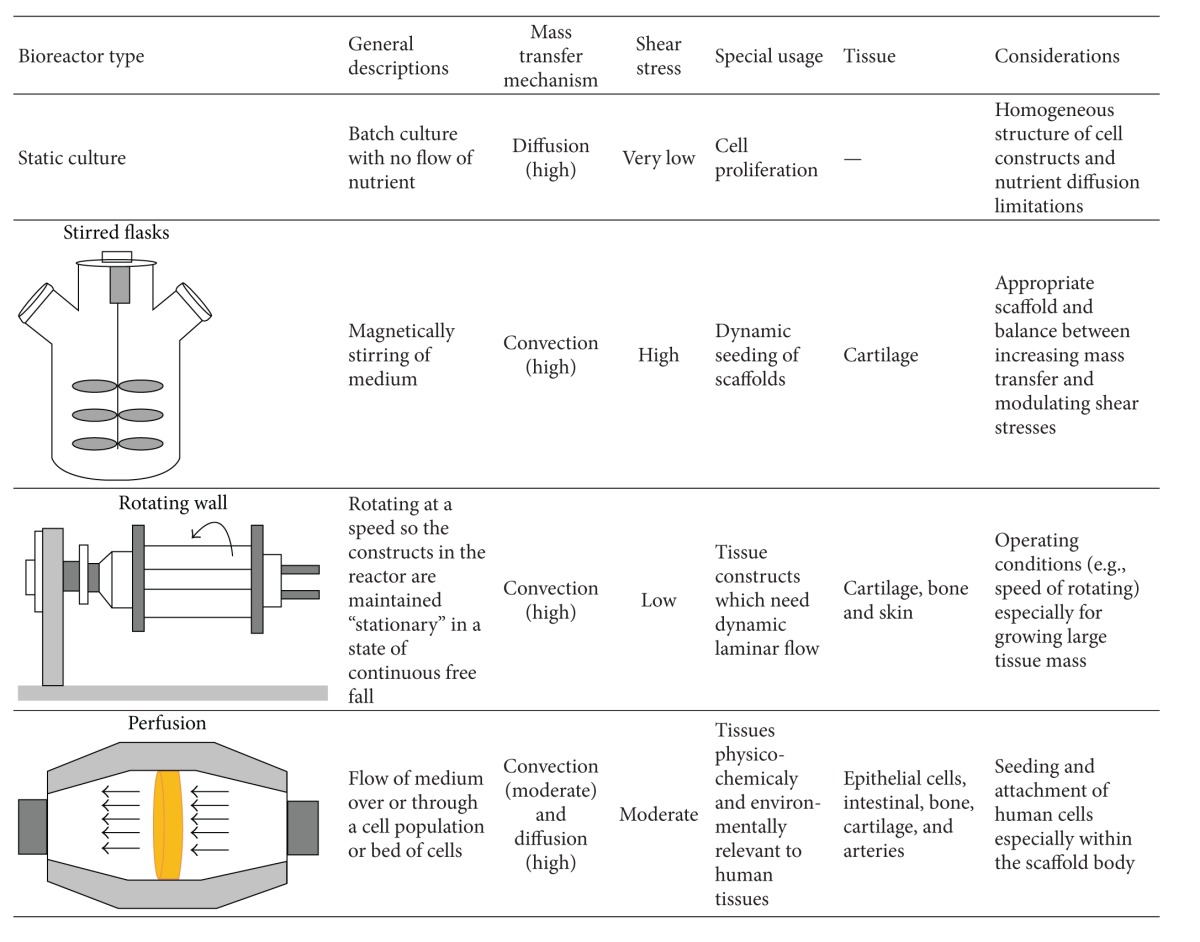

**Table 2:** Methods of measuring oxygen transfer rate.

Measurement method	Basis of the method	Pros	Cons	Ref.
Sulfite oxidation method	Monitoring pH changes during the oxidation of sodium sulfite to sodium sulfate controlled by oxygen depletion rate	(i) Simple and low cost (ii) Can be used for the determination of the interfacial area between gas and liquid (iii) Being accurate for minivolumes of less than 1 mL	(i) The kinetics of the homogeneous catalytic chemical reaction should be known (ii) Limited accuracy by visually determination of color change (iii) High salt concentration (usually 0.5 mol · L^−1^) reduces the maximum solubility of oxygen (iv) Not appropriate in large scale bioreactors (v) High surface tension causes the underestimation of potentially achievable OTR	[72–75]

Dynamic method	Monitoring the dissolved O_2_ concentration during the aeration of the system	(i) Consistent measurement (ii) Does not depend on a zero or reference measurement	(i) Requiring a rapidly responsive, sterilizable, dissolved oxygen probe (ii) Limited application for minititerplates (MTPs) (iii) Not costly favorable	[76, 77]

Optical method	Monitoring the color changes during the sulfite oxidation reaction using a pH sensitive dye (e.g., bromothymol blue)	No need for pH electrode which frequently disturbs the hydrodynamics	Not accurate due to being time dependent of the color shift which indicated the time of the oxidation reaction	[78–80]

Gassing-out method	Monitoring *k*_*L*_ *a* by direct measurement of the rate of increasing dissolved oxygen concentration, after neutralizing the system by flushing nitrogen through the vessel to achieve an oxygen-free solution	(i) Can be applied to different media (for investigating the effect of media composition on oxygen mass transfer) (ii) Does not involve chemical reactions that could impact the measurement precision and the liquid film resistance	A nonrespiring system which is not in exact correspondence to real culturing conditions	[28, 81, 82]

RAMOS (intermittent online) method	Monitoring OTR by periodically repeating an automated measuring cycle composed of a measuring phase and a rinsing phase	(i) Online monitoring system (ii) Can be used simultaneously for measuring OTR in 6–12 parallel vessels	(i) Large amount of sample required for measuring (ii) Not applicable for small volumes (microliters)	[83–85]

Exhaust gas analyzer (continuous online) method	Calculating the OTR by specifying the oxygen concentration difference between the inlet gas stream (O_2_, in) and the outlet gas stream (O_2_, out) using magnetomechanical exhaust gas analyzer (EGA)	(i) Continuous method (ii) Can be used to measure OTR in one to five parallel culture vessels	Only applicable in high volume bioreactors	[86, 87]

Respirometer (offline) method	Measuring of decreasing dissolved oxygen concentration with time after aerating the culture vessel	Can be used for bioreactors of any shape	Difficult manual handling	[88, 89]

**Table 3:** Methods of measuring flow.

Measurement method	Basis of the method	Pros	Cons	Ref.
Particle image velocimetry (PIV), including micro-PIV (*μ*PIV)	Monitoring the displacement of small seeded particles in a region of interest of fluid medium via double-pulsed laser beam	(i) Can be used through an *in vitro *investigation (ii) Noninvasive method (iii) High spatial resolution (iv) Simultaneously determination of velocities of two different phases without disturbing the flow	(i) Almost impossible for *in vivo *experiments (ii) Requiring undistorted optical access to the area of interest for both an excitation laser and an imaging system (iii) Limited of temporal resolution (iv) Requiring appropriate particles to eliminate the differences between solid particles and local fluid velocities	[90–94]

Holographic PIV (HPIV)	Record the particle image field using a reference beam to project the hologram, followed by a 2D plane detector moved through the projected hologram	(i) Can also record a 3D instantaneous flow field (ii) Being user friendly	(i) Reduction of speckle noise (ii) Not handling huge quantities of data (iii) Cannot extract 3D velocity in presence of large gradients/fluctuations (iv) Complexity of system (v) Requiring large depth of focus that affects the measurement accuracy	[93–96]

Particle tracking velocity (PTV)	Measuring particle velocities using video camera recording	(i) Easily determination of even small displacement of particles without confusing them with neighboring ones (ii) Measurement of velocity at the location of a particle, without requiring an averaging over a grid (compared to PIV)	(i) Requiring many individual particles to be reconstructed in space and identified in successive frames (ii) Lower spatial resolution (iii) Time consuming	[95–98]

Laser Doppler anemometry (LDA) or laser Doppler velocimetry (LDV)	Measuring of scattered laser light by particles that pass through a series of interference fringes (a pattern of light and dark surfaces)	(i) High spatial and temporal resolution (typically in the order of 1 kHz) (ii) Nonintrusive method (iii) No calibration required (iv) Recording one, two, or three velocity components simultaneously (v) Also applicable in reversing flows	(i) Cannot simultaneously measure the velocities of different phases (ii) Time consuming (iii) Difficult to analyzing the discrete data stream from the flow	[94–97, 99]

Acoustic Doppler velocimeter (ADV)	Measuring the velocity of particles in a remote sampling volume based on the Doppler shift effect using one transmitter and three receivers	Simultaneously recording nine values with each sample: three velocity components, three signal strength values, and three correlation values	(i) Only suitable for flow conditions with relatively low turbulence level (ii) Required postprocessing of data	[99, 100]

Holographic correlation velocimetry (HCV)	Measuring 3D velocity fields of a fluid at high speed combining a correlation-based approach with in-line holography	(i) Very efficient with regard to the use of light, as it does not rely on side scattering (ii) Very high quality system at a modest cost (iii) Appropriate for high-speed flows and low exposure times (iv) Simple calibration (v) Using relatively low powered lasers (vi) Direct measurement of the velocity field at all depth locations (vii) Nonintrusive technique especially in cell culture procedures	Requiring a separate method to extract velocity data from holographic images	[94, 95]

## References

[1] Pörtner R, Nagel-Heyer S, Goepfert C, Adamietz P, Meenen NM (2005). Bioreactor design for tissue engineering. *Journal of Bioscience and Bioengineering*.

[2] Ellis M, Jarman-Smith M, Chaudhuri JB, Al-Rubeai M, Chaudhuri JB (2005). Bioreactor systems for tissue engineering: a four-dimensional challenge. *Bioreactors For Tissue Engineering: Principles, Design and Operation*.

[3] Sengers BG, Taylor M, Please CP, Oreffo ROC (2007). Computational modelling of cell spreading and tissue regeneration in porous scaffolds. *Biomaterials*.

[4] Radisic M, Park H, Vunjak-Novakovic G, Lanza R, Langer R, Vacanti JP (2008). Cardiac-tissue engineering. *Principles of Tissue Engineering*.

[5] Pancrazio JJ, Wang F, Kelley CA (2007). Enabling tools for tissue engineering. *Biosensors and Bioelectronics*.

[6] Freshney RI, Obradovic B, Grayson W, Cannizzaro C, Vunjak-Novakovic G, Lanza R, Langer R, Vacanti JP (2008). Principles of tissue culture and bioreactor design. *Principles of Tissue Engineering*.

[7] Yang YI, Seol DL, Kim HI, Cho MH, Lee SJ (2007). Continuous perfusion culture for generation of functional tissue-engineered soft tissues. *Current Applied Physics*.

[8] Pörtner R, Giese C, Vunjack-Novakovic G, Ian Freshney R (2006). An overview on bioreactor design, prototyping and process control for reproducible three-dimensional tissue culture. *Culture of Cells For Tissue Engineering*.

[9] Martin I, Wendt D, Heberer M (2004). The role of bioreactors in tissue engineering. *Trends in Biotechnology*.

[10] Martin Y, Vermette P (2005). Bioreactors for tissue mass culture: design, characterization, and recent advances. *Biomaterials*.

[11] El Haj AJ, Wood MA, Thomas P, Yang Y (2005). Controlling cell biomechanics in orthopaedic tissue engineering and repair. *Pathologie Biologie*.

[12] Bueno EM, Bilgen B, Carrier RL, Barabino GA (2004). Increased rate of chondrocyte aggregation in a wavy-walled bioreactor. *Biotechnology and Bioengineering*.

[13] Chen H-C, Hu Y-C (2006). Bioreactors for tissue engineering. *Biotechnology Letters*.

[14] Tabesh H, Amoabediny G, Nik NS (2009). The role of biodegradable engineered scaffolds seeded with Schwann cells for spinal cord regeneration. *Neurochemistry International*.

[15] Kannan RY, Salacinski HJ, Sales K, Butler P, Seifalian AM (2005). The roles of tissue engineering and vascularisation in the development of micro-vascular networks: a review. *Biomaterials*.

[16] Lawrence BJ, Devarapalli M, Madihally SV (2009). Flow dynamics in bioreactors containing tissue engineering scaffolds. *Biotechnology and Bioengineering*.

[17] Wendt D, Timmins N, Malda J, Janssen F, Ratcliffe A, Vunjak-Novakovic G, van Blitterswijk C, Thomsen P, Hubbell J, Cancedda R, de Bruijn JD, Lindahl A (2008). Bioreactors for tissue engineering. *Tissue Engineering*.

[18] Rolfe P (2006). Sensing in tissue bioreactors. *Measurement Science and Technology*.

[19] Muschler GF, Nakamoto C, Griffith LG (2004). Engineering principles of clinical cell-based tissue engineering. *Journal of Bone and Joint Surgery A*.

[20] Wang SJ, Zhong JJ, Yang ST (2007). Bioreactor engineering. *Bioprocessing For Value-Added Products From Renewable Resources*.

[21] Depprich R, Handschel J, Wiesmann H-P, Jäsche-Meyer J, Meyer U (2008). Use of bioreactors in maxillofacial tissue engineering. *British Journal of Oral and Maxillofacial Surgery*.

[22] Eibl R, Eibl D, Portner R, Catapano G, Czermak P (2008). *Cell and Tissue Reaction Engineering*.

[23] Rivera-Solorio I, Kleis SJ (2006). Model of the mass transport to the surface of animal cells cultured in a rotating bioreactor operated in micro gravity. *Biotechnology and Bioengineering*.

[24] Malda J, Radisic M, Levenberg S, van Blitterswijk C, Thomsen P, Hubbell J (2008). Cell nutrition. *Tissue Engineering*.

[25] Garcia-Ochoa F, Gomez E (2009). Bioreactor scale-up and oxygen transfer rate in microbial processes: an overview. *Biotechnology Advances*.

[26] Hermann R, Lehmann M, Büchs J (2003). Characterization of gas-liquid mass transfer phenomena in microtiter plates. *Biotechnology and Bioengineering*.

[27] Suresh S, Srivastava VC, Mishra IM (2009). Techniques for oxygen transfer measurement in bioreactors: a review. *Journal of Chemical Technology and Biotechnology*.

[28] Marques DAV, Torres BR, Porto ALF, Pessoa-Júnior A, Converti A (2009). Comparison of oxygen mass transfer coefficient in simple and extractive fermentation systems. *Biochemical Engineering Journal*.

[29] Tabesh H, Amoabediny G, Salehi-Nik N, Esfahani K, Derakhshanfar H, Zandieh Doulabi B (2010). Use of computerized simulation of engineering parameters in tissue-engineering bioreactors. *European Spine Journal*.

[30] Yeatts AB, Fisher JP (2011). Bone tissue engineering bioreactors: dynamic culture and the influence of shear stress. *Bone*.

[31] Sakamoto N, Saito N, Han X, Ohashi T, Sato M (2010). Effect of spatial gradient in fluid shear stress on morphological changes in endothelial cells in response to flow. *Biochemical and Biophysical Research Communications*.

[32] Simmers MB, Pryor AW, Blackman BR (2007). Arterial shear stress regulates endothelial cell-directed migration, polarity, and morphology in confluent monolayers. *The American Journal of Physiology*.

[33] McCoy RJ, O’Brien FJ (2010). Influence of shear stress in perfusion bioreactor cultures for the development of three-dimensional bone tissue constructs: a review. *Tissue Engineering B*.

[34] Waldman SD, Couto DC, Grynpas MD, Pilliar RM, Kandel RA (2007). Multi-axial mechanical stimulation of tissue engineered cartilage: review. *European Cells and Materials*.

[35] Bacabac RG, Smit TH, Van Loon JJWA, Doulabi BZ, Helder M, Klein-Nulend J (2006). Bone cell responses to high-frequency vibration stress: does the nucleus oscillate within the cytoplasm?. *FASEB Journal*.

[36] Lemon G, King JR, Byrne HM, Jensen OE, Shakesheff KM (2006). Mathematical modelling of engineered tissue growth using a multiphase porous flow mixture theory. *Journal of Mathematical Biology*.

[37] Vatsa A, Smit TH, Klein-Nulend J (2007). Extracellular NO signalling from a mechanically stimulated osteocyte. *Journal of Biomechanics*.

[38] Bacabac RG, Mizuno D, Schmidt CF (2008). Round versus flat: bone cell morphology, elasticity, and mechanosensing. *Journal of Biomechanics*.

[39] Henzler HJ (2000). Particle stress in bioreactors. *Advances in Biochemical Engineering/Biotechnology*.

[40] Zoro BJH, Owen S, Drake RAL, Hoare M (2008). The impact of process stress on suspended anchorage-dependent mammalian cells as an indicator of likely challenges for regenerative medicines. *Biotechnology and Bioengineering*.

[41] Bayati V, Sadeghi Y, Shokrgozar MA (2011). The evaluation of cyclic uniaxial strain on myogenic differentiation of adipose-derived stem cells. *Tissue and Cell*.

[42] Hatami J, Tafazzoli-Shadpour M, Haghighipour N, Shokrgozar MA (2010). Evaluation of Effects of cyclic loading on structural properties of cultured endothelial cell. *Modares Journal of Medical Sciences*.

[43] Haghighipour N, Tafazzoli-Shadpour M, Shokrgozar MA, Amini S, Amanzadeh A, Khorasani MT (2007). Topological remodeling of cultured endothelial cells by characterized cyclic strains. *MCB Molecular and Cellular Biomechanics*.

[44] Haghighipour N, Tafazzoli-Shadpour M, Avolio A (2010). Residual stress distribution in a lamellar model of the arterial wall. *Journal of Medical Engineering and Technology*.

[45] Haghighipour N, Tafazzoli-Shadpour M, Shokrgozar MA, Amini S (2010). Effects of cyclic stretch waveform on endothelial cell morphology using fractal analysis. *Artificial Organs*.

[46] Safshekan F, Tafazzoli Shadpour M, Shokrgozar MA, Haghighipour N, Mahdian R, Hemmati A (2012). Intermittent hydrostatic pressure enhances growth factor-induced chondroinduction of human adipose-derived mesenchymal stem cells. *Artificial Organs*.

[47] Bakker AD, Soejima K, Klein-Nulend J, Burger EH (2001). The production of nitric oxide and prostaglandin E2 by primary bone cells is shear stress dependent. *Journal of Biomechanics*.

[48] ] Kaur H, Carriveau R, Mutus B (2012). A simple parallel plate flow chamber to study effects of shear stress on endothelial cells. *The American Journal of Biomedical Sciences*.

[49] Kang H, Fan Y, Deng X (2011). Vascular smooth muscle cell glycocalyx modulates shear-induced proliferation, migration, and NO production responses. *The American Journal of Physiology*.

[50] Isenberg BC, Williams C, Tranquillo RT (2006). Small-diameter artificial arteries engineered in vitro. *Circulation Research*.

[51] Diamantouros SE, Hurtado-Aguilar LG, Schmitz-Rode T, Mela P, Jockenhoevel S (2013). Pulsatile perfusion bioreactor system for durability testing and compliance estimation of tissue engineered vascular grafts. *Annals of Biomedical Engineering*.

[52] Hahn MS, McHale MK, Wang E, Schmedlen RH, West JL (2007). Physiologic pulsatile flow bioreactor conditioning of poly(ethylene glycol)-based tissue engineered vascular grafts. *Annals of Biomedical Engineering*.

[53] Zaucha MT, Raykin J, Wan W (2009). A novel cylindrical biaxial computer-controlled bioreactor and biomechanical testing device for vascular tissue engineering. *Tissue Engineering A*.

[54] Haghighipour N, Heidarian S, Shokrgozar MA, Amirizadeh N (2012). Differential effects of cyclic uniaxial stretch on human mesenchymal stem cell into skeletal muscle cell. *Cell Biology International*.

[55] Petrović M, Mitraković D, Bugarski B, Vonwil D, Martin I, Obradović B (2009). A novel bioreactor with mechanical stimulation for skeletal tissue engineering. *Chemical Industry and Chemical Engineering Quarterly*.

[56] Tandon N, Marsano A, Maidhof R (2010). Surface-patterned electrode bioreactor for electrical stimulation. *Lab on a Chip*.

[57] Tandon N, Marsano A, Cannizzaro C, Voldman J, Vunjak-Novakovic G (2008). Design of electrical stimulation bioreactors for cardiac tissue engineering. *Proceedings of the Annual International Conference of the IEEE Engineering in Medicine and Biology Society*.

[58] Radisic M, Park H, Shing H (2004). Functional assembly of engineered myocardium by electrical stimulation of cardiac myocytes cultured on scaffolds. *Proceedings of the National Academy of Sciences of the United States of America*.

[59] Tandon N, Marsano A, Maidhof R, Wan L, Park H, Vunjak-Novakovic G (2011). Optimization of electrical stimulation parameters for cardiac tissue engineering. *Journal of Tissue Engineering and Regenerative Medicine*.

[60] Liao IC, Liu JB, Bursac N, Leong KW (2008). Effect of electromechanical stimulation on the maturationofmyotubes on aligned electrospun fibers. *Cellular and Molecular Bioengineering*.

[61] Malda J, Woodfield TBF, van der Vloodt F (2005). The effect of PEGT/PBT scaffold architecture on the composition of tissue engineered cartilage. *Biomaterials*.

[62] Fernandes-Platzgummer A, Diogo MM, Baptista RP, Silva CLD, Cabral JMS (2011). Scale-up of mouse embryonic stem cell expansion in stirred bioreactors. *Biotechnology Progress*.

[63] Partap S, Plunkett NA, O’ Brien FJ, Eberli D (2010). Bioreactors in tissue engineering. *Tissue Engineering*.

[64] Yeatts AB, Fisher JP (2011). Bone tissue engineering bioreactors: dynamic culture and the influence of shear stress. *Bone*.

[65] Oragui E, Nannaparaju M, Khan WS (2011). The role of bioreactors in tissue engineering for musculoskeletal applications. *The Open Orthopaedics Journal*.

[66] Zhang X, Bürki C-A, Stettler M (2009). Efficient oxygen transfer by surface aeration in shaken cylindrical containers for mammalian cell cultivation at volumetric scales up to 1000 L. *Biochemical Engineering Journal*.

[67] Belfiore LA, Bonani W, Leoni M, Belfiore CJ (2009). Pressure-sensitive nutrient consumption via dynamic normal stress in rotational bioreactors. *Biophysical Chemistry*.

[68] Nesic D, Whiteside R, Brittberg M, Wendt D, Martin I, Mainil-Varlet P (2006). Cartilage tissue engineering for degenerative joint disease. *Advanced Drug Delivery Reviews*.

[69] Khetani SR, Bhatia SN (2006). Engineering tissues for in vitro applications. *Current Opinion in Biotechnology*.

[70] Yu X, Botchwey EA, Levine EM, Pollack SR, Laurencin CT (2004). Bioreactor-based bone tissue engineering: the influence of dynamic flow on osteoblast phenotypic expression and matrix mineralization. *Proceedings of the National Academy of Sciences of the United States of America*.

[71] Zhang Z-Y, Teoh SH, Chong W-S (2009). A biaxial rotating bioreactor for the culture of fetal mesenchymal stem cells for bone tissue engineering. *Biomaterials*.

[72] Lotter S, Büchs J (2004). Utilization of specific power input measurements for optimization of culture conditions in shaking flasks. *Biochemical Engineering Journal*.

[73] Anderlei T, Zang W, Papaspyrou M, Büchs J (2004). Online respiration activity measurement (OTR, CTR, RQ) in shake flasks. *Biochemical Engineering Journal*.

[74] Freyer SA, König M, Künkel A (2004). Validating shaking flasks as representative screening systems. *Biochemical Engineering Journal*.

[75] Akgün A, Müller C, Engmann R, Büchs J (2008). Application of an improved continuous parallel shaken bioreactor system for three microbial model systems. *Bioprocess and Biosystems Engineering*.

[76] Jamnongwong M, Loubiere K, Dietrich N, Hébrard G (2010). Experimental study of oxygen diffusion coefficients in clean water containing salt, glucose or surfactant: consequences on the liquid-side mass transfer coefficients. *Chemical Engineering Journal*.

[77] Bellucci JJ, Hamaker KH (2011). Evaluation of oxygen transfer rates in stirred-tank bioreactors for clinical manufacturing. *Biotechnology Progress*.

[78] Duetz WA, Witholt B (2004). Oxygen transfer by orbital shaking of square vessels and deepwell microtiter plates of various dimensions. *Biochemical Engineering Journal*.

[79] Doig SD, Pickering SCR, Lye GJ, Baganz F (2005). Modelling surface aeration rates in shaken microtitre plates using dimensionless groups. *Chemical Engineering Science*.

[80] Therning P, Rasmuson A (2006). Mass transfer measurements in a non-isothermal bubble column using the uncatalyzed oxidation of sulphite to sulphate. *Chemical Engineering Journal*.

[81] Cascaval D, Galaction A-I, Folescu E, Turnea M (2006). Comparative study on the effects of n-dodecane addition on oxygen transfer in stirred bioreactors for simulated, bacterial and yeasts broths. *Biochemical Engineering Journal*.

[82] Puthli MS, Rathod VK, Pandit AB (2005). Gas-liquid mass transfer studies with triple impeller system on a laboratory scale bioreactor. *Biochemical Engineering Journal*.

[83] Seletzky JM, Noack U, Fricke J, Hahn S, Büchs J (2006). Metabolic activity of Corynebacterium glutamicum grown on L-lactic acid under stress. *Applied Microbiology and Biotechnology*.

[84] Amoabediny G, Abbas MPH, Büchs J (2010). Determination of CO_2_ sensitivity of micro-organisms in shaken bioreactors. II. Novel online monitoring method. *Biotechnology and Applied Biochemistry*.

[85] Amoabediny G, Büchs J (2010). Determination of CO_2_ sensitivity of micro-organisms in shaken bioreactors. I. Novel method based on the resistance of sterile closure. *Biotechnology and Applied Biochemistry*.

[86] Seletzky JM, Noack U, Hahn S, Knoll A, Amoabediny G, Büchs J (2007). An experimental comparison of respiration measuring techniques in fermenters and shake flasks: exhaust gas analyzer vs. RAMOS device vs. respirometer. *Journal of Industrial Microbiology and Biotechnology*.

[87] Peña C, Peter CP, Büchs J, Galindo E (2007). Evolution of the specific power consumption and oxygen transfer rate in alginate-producing cultures of Azotobacter vinelandii conducted in shake flasks. *Biochemical Engineering Journal*.

[88] Scheidle M, Klinger J, Büchs J (2007). Combination of on-line pH and oxygen transfer rate measurement in shake flasks by fiber optical technique and respiration activity monitoring system (RAMOS). *Sensors*.

[89] Ortigara ARC, Foladori P, Andreottola G (2011). Kinetics of heterotrophic biomass and storage mechanism in wetland cores measured by respirometry. *Water Science and Technology*.

[90] Liovic P, Šutalo ID, Stewart R, Glattauer V, Meagher L Fluid flow and stresses on microcarriers in spinner flask bioreactors.

[91] Rossi M, Lindken R, Hierck B P, Westerweel J Microfluidic system for the study of mechanical and biochemical response of endothelial cells to flow-induced mechanical stimuli.

[92] Leong Ch M, Voorhees A, Nackman T Wei GB (2013). Flow bioreactor design for quantitative measurements over endothelial cells using micro-particle image velocimetry. *Review of Scientific Instruments*.

[93] Nguyen CV, Carberry J, Fouras A (2011). Volumetric-correlation PIV to measure particle concentration and velocity of microflows. *Experiments in Fluids*.

[94] Higgins SPA, Samarage CR, Paganin DM, Fouras A Holographic Correlation Velocimetry.

[95] Ismadi MZ, Higgins S, Samarage CR, Paganin D, Hourigan K (2013). Optimisation of a stirred bioreactor through the use of a novel holographic correlation velocimetry flow measurement technique. *Plos ONE*.

[96] Ooms T, Koek W, Westerweel J (2008). Digital holographic particle image velocimetry: eliminating a sign-ambiguity error and a bias error from the measured particle field displacement. *Measurement Science and Technology*.

[97] Deen NG, Hjertager BH, Solberg T Comparison of PIV and LDA measurement methods applied to the gas-liquid flow in a bubble column.

[98] Feng Y, Goree J, Liu B (2011). Errors in particle tracking velocimetry with high-speed cameras. *Review of Scientific Instruments*.

[99] Chara Z, Matousek V Comparative study of ADV and LDA measuring techniques.

[100] Chanson H, Trevethan M, Aoki SI Acoustic Doppler velocimetry (ADV) in a small estuarine system.

[101] Abousleiman RI, Sikavitsas VI (2006). Bioreactors for tissues of the musculoskeletal system. *Advances in Experimental Medicine and Biology*.

[102] Cimetta E, Flaibani M, Mella M (2007). Enhancement of viability of muscle precursor cells on 3D scaffold in a perfusion bioreactor. *International Journal of Artificial Organs*.

[103] Kim SS, Penkala R, Abrahimi P (2007). A perfusion bioreactor for intestinal tissue engineering. *Journal of Surgical Research*.

[104] Sikavitsas VI, Bancroft GN, Lemoine JJ, Liebschner MAK, Dauner M, Mikos AG (2005). Flow perfusion enhances the calcified matrix deposition of marrow stromal cells in biodegradable nonwoven fiber mesh scaffolds. *Annals of Biomedical Engineering*.

[105] Lovett M, Rockwood D, Baryshyan A, Kaplan DL (2010). Simple modular bioreactors for tissue engineering: a system for characterization of oxygen gradients, human mesenchymal stem cell differentiation, and prevascularization. *Tissue Engineering C*.

[106] Lee C, Grad S, Wimmer M, Alini M, Ashammakhi N, Reis RL (2005). The influence of mechanical stimuli on articular cartilage tissue engineering. *Topics in Tissue Engineering*.

[107] Cerulli J (2011). *Perfusion Bioreactor for the Development of Tissue-Engineered Blood Vessels [Bachelor thesis]*.

[108] Radisic M, Marsano A, Maidhof R, Wang Y, Vunjak-Novakovic G (2008). Cardiac tissue engineering using perfusion bioreactor systems. *Nature Protocols*.

[109] Meyer U, Büchter A, Nazer N, Wiesmann HP (2006). Design and performance of a bioreactor system for mechanically promoted three-dimensional tissue engineering. *British Journal of Oral and Maxillofacial Surgery*.

[110] Chung CA, Chen CP, Lin TH, Tseng CS (2008). A compact computational model for cell construct development in perfusion culture. *Biotechnology and Bioengineering*.

[111] Chung CA, Yang CW, Chen CW (2006). Analysis of cell growth and diffusion in a scaffold for cartilage tissue engineering. *Biotechnology and Bioengineering*.

[112] Lewis MC, MacArthur BD, Malda J, Pettet G, Please CP (2005). Heterogeneous proliferation within engineered cartilaginous tissue: the role of oxygen tension. *Biotechnology and Bioengineering*.

[113] Malda J, Rouwkema J, Martens DE (2004). Oxygen gradients in tissue-engineered PEGT/PBT cartilaginous constructs: measurement and modeling. *Biotechnology and Bioengineering*.

[114] Yan X, Bergstrom DJ, Chen XB (2012). Modeling of cell cultures in perfusion bioreactors. *IEEE Transactions on Biomedical Engineering*.

[115] O’Dea RD, Waters SL, Byrne HM (2008). A two-fluid model for tissue growth within a dynamic flow environment. *European Journal of Applied Mathematics*.

[116] Shi Y (2008). Numerical simulation of global hydro-dynamics in a pulsatile bioreactor for cardiovascular tissue engineering. *Journal of Biomechanics*.

[117] Sucosky P, Osorio DF, Brown JB, Neitzel GP (2004). Fluid mechanics of a spinner-flask bioreactor. *Biotechnology and Bioengineering*.

[118] Shakeel M (2011). *Continuum modelling of cell growth andnutrient transport in a perfusion bioreactor [Ph.D. thesis]*.

[119] Sengers BG, van Donkelaar CC, Oomens CWJ, Baaijens FPT (2005). Computational study of culture conditions and nutrient supply in cartilage tissue engineering. *Biotechnology Progress*.

[120] Chung CA, Chen CW, Chen CP, Tseng CS (2007). Enhancement of cell growth in tissue-engineering constructs under direct perfusion: modeling and simulation. *Biotechnology and Bioengineering*.

[121] Coletti F, Macchietto S, Elvassore N (2006). Mathematical modeling of three-dimensional cell cultures in perfusion bioreactors. *Industrial and Engineering Chemistry Research*.

[122] Whittaker RJ, Booth R, Dyson R (2009). Mathematical modelling of fibre-enhanced perfusion inside a tissue-engineering bioreactor. *Journal of Theoretical Biology*.

[123] Amoabediny G, Büchs J (2007). Modelling and advanced understanding of unsteady-state gas transfer in shaking bioreactors. *Biotechnology and Applied Biochemistry*.

[124] Anderlei T, Mrotzek C, Bartsch S, Amoabediny G, Peter CP, Büchs J (2007). New method to determine the mass transfer resistance of sterile closures for shaken bioreactors. *Biotechnology and Bioengineering*.

[125] Pisu M, Lai N, Cincotti A, Concas A, Cao G (2004). Modeling of engineered cartilage growth in rotating bioreactors. *Chemical Engineering Science*.

[126] Abdullah NS, Das DB (2007). Modelling nutrient transport in hollow fibre membrane bioreactor for growing bone tissue with consideration of multi-component interactions. *Chemical Engineering Science*.

[127] Yu P, Lee TS, Zeng Y, Low HT (2007). A 3D analysis of oxygen transfer in a low-cost micro-bioreactor for animal cell suspension culture. *Computer Methods and Programs in Biomedicine*.

[128] Cioffi M, Boschetti F, Raimondi MT, Dubini G (2006). Modeling evaluation of the fluid-dynamic microenvironment in tissue-engineered constructs: a micro-CT based model. *Biotechnology and Bioengineering*.

[129] Hutmacher DW, Singh H (2008). Computational fluid dynamics for improved bioreactor design and 3D culture. *Trends in Biotechnology*.

[130] Porter B, Zauel R, Stockman H, Guldberg R, Fyhrie D (2005). 3-D computational modeling of media flow through scaffolds in a perfusion bioreactor. *Journal of Biomechanics*.

[131] Dubey H, Das SK, Panda T (2006). Numerical simulation of a fully baffled biological reactor: the differential circumferential averaging mixing plane approach. *Biotechnology and Bioengineering*.

[132] Singh H, Eng SA, Lim TT, Hutmacher DW (2007). Flow modeling in a novel non-perfusion conical bioreactor. *Biotechnology and Bioengineering*.

[133] Zeng Y, Lee T-S, Yu P, Roy P, Low H-T (2006). Mass transport and shear stress in a microchannel bioreactor: numerical simulation and dynamic similarity. *Journal of Biomechanical Engineering*.

[134] Provin C, Takano K, Sakai Y, Fujii T, Shirakashi R (2008). A method for the design of 3D scaffolds for high-density cell attachment and determination of optimum perfusion culture conditions. *Journal of Biomechanics*.

[135] Boschetti F, Raimondi MT, Migliavacca F, Dubini G (2006). Prediction of the micro-fluid dynamic environment imposed to three-dimensional engineered cell systems in bioreactors. *Journal of Biomechanics*.

[136] Pouran B, Amoabediny G, Saghafinia S, Haji Abbas MP (2012). Characterization of interfacial hydrodynamics in a single cell of shaken microtiter plate bioreactors applying computational fluid dynamics technique. *Procedia Engineering*.

[137] Yu P, Lee TS, Zeng Y, Low HT (2005). Fluid dynamics of a micro-bioreactor for tissue engineering. *Fluid Dynamics and Materials Processing*.

[138] Bilgen B, Barabino GA (2007). Location of scaffolds in bioreactors modulates the hydrodynamic environment experienced by engineered tissues. *Biotechnology and Bioengineering*.

[139] Tabrizi HO, Amoabediny G, Moshiri B (2011). Novel dynamic model for aerated shaking bioreactors. *Biotechnology and Applied Biochemistry*.

